# Comparing logistic regression, support vector machines, and permanental classification methods in predicting hypertension

**DOI:** 10.1186/1753-6561-8-S1-S96

**Published:** 2014-06-17

**Authors:** Hsin-Hsiung Huang, Tu Xu, Jie Yang

**Affiliations:** 1 Department of Statistics, University of Central Florida, Orlando, FL 32816-2370, USA

## Abstract

In this paper, we compare logistic regression and 2 other classification methods in predicting hypertension given the genotype information. We use logistic regression analysis in the first step to detect significant single-nucleotide polymorphisms (SNPs). In the second step, we use the significant SNPs with logistic regression, support vector machines (SVMs), and a newly developed permanental classification method for prediction purposes. We also detect rare variants and investigate their impact on prediction. Our results show that SVMs and permanental classification both outperform logistic regression, and they are comparable in predicting hypertension status.

## Background

Genetic Analysis Workshop 18 (GAW18) data provide genotypes from a real human whole-genome sequencing study including systolic blood pressure (SBP) and diastolic blood pressure (DBP), as well as covariates such as age, medication use, cigarette smoking, parents, and pedigrees. The genome-wide association study (GWAS) data of 1043 individuals come from 20 Mexican American pedigrees enriched for type 2 diabetes from San Antonio, Texas. The data are longitudinal, with 3 measurements for most participants at 4 time intervals (1981 to 1996, 1997 to 2000, 1998 to 2006, and 2009 to 2011). Because there are missing observations in the original phenotype data, simulated hypertension status and blood pressure data sets were generated according to the real genotypes and other covariates.

In our analysis, we use the GWAS data for chromosome 3 and the simulated phenotype data from GAW18. The GWAS of chromosome 3 contains 65,519 single-nucleotide polymorphisms (SNPs). Simulated phenotypes were generated from the real data, which consist of 849 individuals and 3 examination times. The sample for the simulated data set is of the 849 individuals who have both phenotypes and imputed sequence data in the real data set. Two hundred replicates of simulated phenotype data are provided. All individuals have simulated phenotype information at 3 time points with no missing data. The goal of our analysis is to predict whether people will have hypertension. Hence, our data set includes a binary response for simulated hypertension status, GWAS genotypes, age, sex, smoking status, parents, and pedigree information. The covariate medication status is excluded because it contains diagnosis information and is thus highly correlated with hypertension. We choose different numbers of SNPs and compare the corresponding prediction error rates. We also compare the performance of 3 approaches including logistic regression analysis, support vector machines (SVMs) [1-3], and the newly developed permanental classification method [4].

## Methods

In this paper, we treat SIMPHEN.1.csv as the training data set and SIMPHEN.2.csv--SIMPHEN.5.csv as the testing sets. The conclusions are similar if we use other replicates as training and testing data sets.

### Single-nucleotide polymorphism selection using logistic regression

Our main goals are to predict hypertension and to compare the prediction performances of logistic regression and the other 2 classification methods. In the GAW18 data, the hypertension diagnosis variable HTN is binary (yes = 1; no = 0). The logistic regression model has been used extensively for handling categorical responses and shows competitive performance in a wide range of applications. We apply it in this paper as a baseline model. Two sources of data are used in the logistic regression analysis: simulated phenotype data and SNP data. We use simulated phenotype data rather than the real phenotypes because the simulated phenotypes do not contain missing values.

In the first step, the baseline logistic regression model is fitted with SIMPHEN.1.csv as *logit(Pr(HTN = 1))= Smoke + Age + Sex + Age × Sex + Mother + Father + Pedigree*.

In this step, we treat the 3 repeated measurements for each participant as 3 observations to increase the power in identifying the effects of 2 time-variant covariates, Age and Smoke. The interaction term Age Sex is also included because of its significance. The factors Father and Mother represent the hypertension status of the parents. The factor Pedigree is included to identify critical effects associated with family history. Father, Mother, and Pedigree are all highly significant in the baseline model.

Next, each SNP in the recommended dataset chr3-gwas.csv is added separately into the model to measure its significance in terms of *p*-values.

logit (Pr(HTN = 1)) = Smoke + Age + Sex + Age × Sex + Mother + Father + Pedigree + SNP_i_

We sort the corresponding *p*-values increasingly and regard SNPs at the beginning of the list as the most significant ones. The SNPs are listed in Table 1. We also perform SNP selections using linear regression analysis with SBP and DBP as response variables separately because HTN = 1 is defined as SBP greater than 140 mm Hg or DBP greater than 90 mm Hg. No transformation is needed for SBP or DBP according to Box-Cox power transformation. We expect that the most significant SNPs for HTN are also ranked high using SBP or DBP. Indeed, rs11711953 and rs11706549 are the 2 most significant SNPs for all 3 responses.

**Table 1:** Most significant 18 single-nucleotide polymorphisms based on logistic regression on SIMPHEN.1.csv and corresponding *p*-values

1	2	3	4	5	6	7	8	9
rs11711953	rs11706549	rs275678	rs9828391	rs7653745	rs6789918	rs9829009	rs11719850	rs7645789

4.4 × 10^−13^	1.1 × 10^−12^	6.8 × 10^−11^	9.9 × 10^−11^	1.2 × 10^−10^	6.4 × 10^−10^	8.4 × 10^−10^	1.5 × 10^−9^	2.0 × 10^−9^

**10**	**11**	**12**	**13**	**14**	**15**	**16**	**17**	**18**

rs7609918	rs6444467	rs1471695	rs7632157	rs17785248	rs6777472	rs16862782	rs12497460	rs4680987

2.1 × 10^−9^	2.2 × 10^−9^	2.2 × 10^−9^	3.0 × 10^−9^	3.0 × 10^−9^	3.5 × 10^−9^	3.7 × 10^−9^	4.3 × 10^−9^	4.7 × 10^−9^

Table 2 lists the 2 × 3 frequency table of hypertension diagnosis and genotype for rs11711953, where XX represents missing values. The *p*-value of the associated chi-square test is 0.0011, which implies a significant difference in genotype frequencies between the hypertension group and the non-hypertension group.

**Table 2:** Frequency table of hypertension status and genotype

rs11711953	CC	TC	XX
No	1858	125	13
Yes	536	13	2

*CC*, cytosine- cytosine pair; *TC*, thymine- thymine pair; *XX*, unknown pair.

Attempts to use a subset of only low linkage disequilibrium (LD) SNPs (67 SNPs with mutual correlation *r *<0.95 and 434 SNPs with *r *<0.99 among the 2,500 most significant SNPs) were less successful than using the entire list. Therefore, in the remainder of the paper, we focus on the entire list with the exception that SNPs in perfect LD (*r *= 1) are removed, which leaves 62,735 SNPs for logistic regression analysis.

### Prediction based on logistic regression

In the second step, we add the most significant SNPs, that is, those SNPs with smallest *p*-values, into the baseline logistic regression model and use the extended model to predict hypertension status with at most four indicator variables for each SNP (the model could become too complex to handle even with a small number of SNPs). To be clear, each SNP provided in the data has up to four genotypes including XX. For each SNP, the insignificant genotypes (*p*-value ≥0.05) are grouped into a single category, -Not Significant (NS).

### Classification

The model is fitted on the training set SIMPHEN.1.csv with the top 5, 10, 15, 20, 50, 100, and 200 SNPs as the predictors and SIMPHEN.2.csv-- SIMPHEN.5.csv as the testing sets. We apply the supervised classification methods of support vector machines and the permanental classification to predicting hypertension.

Given a training data set, $\left\{\left(x_{i},y_{i}\right)|x_{i}\in {ℜ}^{p},y_{i}\in \left\{0,1\right\}\right\}$, where the $y_{i}$ indicates the class to which the covariate $x_{i}$ belongs, SVMs use a projection function of the input data into a high-dimensional feature space in which a hyperplane with the maximal margin is found to divide the observations having $y_{i}=0$ from those having $y_{i}=1$. The testing sets are then mapped into that same space and predicted to be in a category based on which side of the hyperplane they fall on. Here we use a radial basis kernel $K\left(x,x^{\prime }\right)=exp\left\{-\gamma x-{x^{\prime }}^{2}\right\}$ with SVM with the parameters (γ,C) where *C *is the size of error penalty. To tune the (γ,C) parameters, we use 10-fold cross-classification on the training data.

The permanental classification method is a novel stochastic classification method. It regards all observations belonging to the same class as a realization of a stochastic point process, called a permanental process. For each class, the method provides a probability of membership by measuring the stochastic distance between the new observation and each class. For our data analysis, we use the covariance function $K\left(x,x^{\prime }\right)=exp\left\{-||x-{x^{\prime }||}^{2}/\tau^{2}\right\}$ and parameter for the permanental process and 10-fold cross-validation to tune (α,τ) on the training data. One of the major advantages of permanental classification is that it is capable of handling high-dimensional data and multiple classes efficiently.

## Results and discussion

### Effect of logistic regression

Given the fitted logistic regression model, the predicted hypertension status is "yes = 1" if $\hat{Pr}\left(HTN=1\right)\geq 0.5$ and "no = 0" otherwise. We then perform the logistic regression with different numbers (5, 10, 15, 20) of non-identical SNPs included into the baseline model. The prediction errors of logistic regression are summarized in Table 3. It can be seen from Table 3 that the decrease in training error is small, as the number of SNPs increases from 5 to 20 while the testing errors increase. This indicates that overfitting becomes an issue when more than 10 SNPs are included. Moreover, among the 20 SNPs added into the model, there are 11 SNPs with mutual correlation less than 0.90 (12 for 0.95 and 15 for 0.99). Prediction error rates are reported in Table 3.

**Table 3:** Prediction errors of logistic regression with multiple single-nucleotide polymorphisms across different data sets and number of single-nucleotide polymorphisms included

Number of SNPs	SIMPHEN.1	SIMPHEN.2	SIMPHEN.3	SIMPHEN.4	SIMPHEN.5
	**Training**	**Testing 1**	**Testing 2**	**Testing 3**	**Testing 4**

0	0.221	0.232	0.223	0.216	0.228
5	0.211	0.229	0.218	0.210	0.228
10	0.189	0.230	0.225	0.207	0.223
15	0.190	0.234	0.224	0.208	0.225
20	0.188	0.242	0.229	0.213	0.235

*SNP*, single-nucleotide polymorphism.

### Rare variants

Rare variants could be critical in interpreting some individual cases in practice [5]. However, it is hard to detect these rare variants using regression models, so we conduct a separate analysis for the rare variants. We define rare variants as genotypes whose minor allele frequency is less than 5% over the whole study group in each SNP. The number of rare variants found is 31,794. Chi-square tests are performed to detect the most significant rare variants for hypertension in terms of *p*-values. Table 4 lists 2 × 2 frequency tables for the top 2 rare variants. The corresponding *p*-values for them are 4.06 × 10^-12 ^and 2.64 × 10^-10^. As with SNPs, many identical rare variants exist. Therefore, different numbers (5, 10, 15, 20) of significant non-identical rare variants are added into the baseline model. The program does not converge if more rare variants are selected. When the 20 most significant rare variants (*p*-value <10^-8^) are included, only 6 rare variants among them have mutual correlation less than 0.99. As a result, in most cases, the prediction errors of models with selected rare variants do not improve much. It is not surprising that rare variants do not work as well as the original SNPs because rare variants help only with the prediction of a small portion of patients.

**Table 4:** Rare variants

HTN/genotype	rs9829721		rs776105	
		
	TT	Non-TT	AA	Non-AA
0	17	1857	58	1816
1	37	636	62	611

*AA*, adenine-adenine pair; *HTN*, hypertension; *TT*, thymine-thymine pair.

### Effect of classification

We use the same genotype (SNPs) and covariates (Smoke, Age, Sex, Age Sex, Mother, Father, Pedigree) chosen by logistic regression. The numbers of SNPs used for SVM and permanental classification are 0, 5, 10, 15, 20, 50, 100, and 200. Tables 5, 6 and 7 list the average prediction error rates of all four testing sets, from the second to the fifth, by using common variants, rare variants, and their combinations. The analysis of the simulated data shows that the best prediction error rates of SVM and permanental classification are both close to 12%. Moreover, the rare variants do not provide significant improvement for prediction.

**Table 5:** Prediction errors of support vector machine and permanental classification using common variants

Number of SNPs	0	5	10	15	20	50	100	200
Radial kernel SVM (training)	0.2301	0.0291	0.0180	0.0203	0.0130	0.0175	0.0052	0.0008
Radial kernel SVM (testing)	0.2419	0.1460	0.1350	0.1303	0.1272	0.1257	0.1213	0.1248
Permanental classification (training)	0.2231	0.1031	0.0971	0.0536	0.0404	0.0334	0.0337	0.0321
Permanental classification (testing)	0.2642	0.1517	0.1433	0.1473	0.1350	0.1347	0.1233	0.1231

*SNP*, single-nucleotide polymorphism; *SVM*, support vector machine.

**Table 6:** Prediction errors of support vector machine and permanental classification using rare variants

Number of SNPs	10	50	100	200
Radial kernel SVM (training)	0.0795	0.0087	0.0087	0.0087
Radial kernel SVM (testing)	0.2484	0.2440	0.2432	0.2424
Permanental classification (training)	0.0843	0.0637	0.0711	0.0575
Permanental classification (testing)	0.2533	0.2330	0.2331	0.2303

*SNP*, single-nucleotide polymorphism; *SVM*, support vector machine.

**Table 7:** Prediction errors of support vector machine and permanental classification using common and rare variants

Number of common and rare variants	(10,10)	(50,50)	(100,100)	(200,200)
Radial kernel SVM (training)	0.0195	0.0067	0.0067	0.0067
Radial kernel SVM (testing)	0.1350	0.1351	0.1350	0.1301
Permanental classification (training)	0.0943	007137	0.0631	0.0513
Permanental classification (testing)	0.1433	0.1330	0.1300	0.1300

*SVM*, support vector machine.

## Conclusions

The logistic regression model is used as a baseline. A sophisticated regression model could be used, but here we focus on the SVM and permanental classification methods. The pedigree and SNP information helps predict hypertension. The strength of SVM and permanental classification is that they are able to handle a lot of strong LD SNPs. When the most significant 20 or fewer SNPs from the single-SNP logistic regression are used as predictors for SVM or permanental classification classifiers, the error rates are comparable. Moreover, the error rates are reduced from about 22% for multi-SNP logistic regression to 12% for SVM and permanental classification, when the most significant 100 SNPs from the single-SNP logistic regression are used as predictors. The testing error rate increases somewhat for SVM, and the testing error rate decreases for permanental classification, when the most significant 200 SNPs are used as predictors. This implies that overfitting occurs for SVM in this situation. The nonparametric SVM and semiparametric permanental classification can include more SNPs and thus can result in lower prediction errors.

To identify significant SNPs, HTN as a binary response may be less powerful than the quantitative blood pressure measurements. We will explore the performance of SNP selection using blood pressure measurements to selection based on HTN in a subsequent paper. If some rare variants do make contributions to hypertension, they may not be able to be identified using regression because of the small group size of rare variants. Moreover, the rare variant provided only small improvements for predicting hypertension. Collapsing methods [6,7] that create dummy variables indicating the presence of every rare variant in a gene can be more powerful, and many different such approaches are in the literature. Based on current testing results, the classification methods outperformed logistic regression because they included a large number of SNPs; the pedigree information and the common variants of SNPs contribute greatly to prediction. In addition, SVM and permanental classification have comparable prediction errors when considering pedigree information.

## Competing interests

The authors declare that they have no competing interests.

## Authors' contributions

TX requested data, performed the logistic regression analysis, and drafted the manuscript. HHH performed the SVM and permanental classification analysis and finished the manuscript. TX and HHH contributed equally. JY advised the research and helped with the statistical analysis and manuscript. All authors read and approved the final manuscript.
